# Antileishmanial Activities of Carvacrol Nanoencapsulate in Biopolymeric Nanoparticles

**DOI:** 10.1002/cbdv.202403341

**Published:** 2025-05-09

**Authors:** Joyce Cordeiro Borges, Anne Beatriz Cunha Barros, Leonardo Lima Cardoso, Tatjana de Lima Souza Keesen, Luís André de Almeida Campos, Isabella Macário Ferro Cavalcanti, Elisângela Afonso de Moura Kretzschmar

**Affiliations:** ^1^ Laboratory of Industrial Nanoscience and Nanobiotechnology (LANNI) Center for Biotechnology Federal University of Paraiba, Castelo Branco João Pessoa Paraiba Brazil; ^2^ Laboratory of Immunology of Infectious Diseases (LABIDIC) Center for Biotechnology Federal University of Paraiba, Castelo Branco João Pessoa Paraiba Brazil; ^3^ Laboratory of Microbiology and Parasitology, Campus Ouricuri University of Pernambuco Ouricuri Pernambuco Brazil; ^4^ Keizo Asami Institute (iLIKA), Federal University of Pernambuco Recife Pernambuco Brazil

**Keywords:** biopolymers, *Leishmania* spp, monoterpenes, nanobiotechnology

## Abstract

Visceral leishmaniasis (VL) is a neglected parasitic disease, and the first‐line treatments for VL include drugs that exhibit serious toxicological issues. In this sense, new molecules are sought for VL treatment, such as Carvacrol (Car), a phenolic monoterpene that has shown strong activity against *Leishmania* spp. However, its low solubility prevents its free administration, requiring a new therapeutic strategy such as encapsulation in chitosan biopolymeric nanoparticles. This study aimed to develop chitosan biopolymeric nanoparticles (NPChi) encapsulating Car (NPCar) and evaluate their in vitro anti‐leishmanial activity. The NPChi had particle sizes of 89.43 ± 0.774 nm, a polydispersity index (PDI) of 0.168 ± 0.01 and zeta potential of 12.8 ± 2.17 mV. The NPCar showed particle size of 144.9 ± 1.7 nm, PDI of 0.224 and zeta potential of 15.7 ± 1.01 mV. NPCar reduced the cytotoxicity of Car on human erythrocytes. Moreover, NPCar showed inhibition of *Leishmania infantum* with an inhibitory concentration (IC_50_) of 2.659 ± 0.26 µg/mL. Thus, NPCar exhibited enhanced anti‐leishmanial activity compared to free Car while reducing cytotoxicity on human erythrocytes, making them promising candidates for further studies on VL treatment.

## Graphical abstract

The study obtained biopolymeric nanoparticles containing carvacrol (NPCar) and evaluated their anti‐leishmanial activity. NPCar obtained presented physicochemical characteristics due to its ideal nanometric size and zeta potential, and low polydispersity index. Our findings indicate the efficacy of carvacrol as a nanotechnological therapeutic approach for the inhibition of the parasite *Leishmania infantum*, which may represent a nanotechnological innovation for the treatment of this visceral leishmaniasis

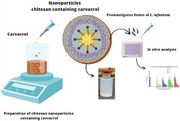

## 1 Introduction

Leishmaniasis is a neglected tropical disease and a major public health concern. It is a disease that has a strong potential for outbreak and mortality. Approximately 700 000 to 1 million new cases are reported worldwide, being fatal if untreated in more than 95% of cases. A total of 90% of new cases reported to the WHO occurred in countries such as Brazil, China, Ethiopia, Eritrea and India [1].

Leishmaniasis is a neglected tropical disease caused by more than 20 *Leishmania* species, transmitted through the bite of infected sandflies (genus *Lutzomyia*). The clinical forms are cutaneous, mucocutaneous and visceral, also known as kala‐azar [2]. Among the clinical forms, the most notable is visceral leishmaniasis (VL), a systemic form of the disease and is characterized by splenomegaly, hepatomegaly, hypergammaglobulinemia and pancytopenia [3]. The protozoa that usually cause VL are the species *L. donovani, L. infantum* and *L. chagasi*, however, Brazil presented more than 97% of VL cases throughout America, with *Leishmania infantum* as the main etiological agent [4].

Despite the availability of treatments, current anti‐leishmanial drugs exhibit severe toxicity, high costs, and emerging parasite resistance, necessitating alternative therapeutic strategies [5]. In this scenario, natural products stand out in the bioprospecting of new bioactive molecules, and among them, some monoterpenes have demonstrated activity against *Leishmania* spp., such as 2‐methyl‐5‐[1‐methyl‐ethyl] phenol known as Car [6]. Car is a phenolic monoterpene isolated from essential oils of plants, such as *Origanum vulgare*, *Thymus vulgaris*, *Lepidium flavum*, and *Citrus aurantium bergamia* [7]. Studies have shown that the biological properties of Car include antimicrobial, anti‐inflammatory, antioxidant, anticancer, immunomodulatory, and other therapeutic properties [8]. The antimicrobial activity of Car has been superior to that of other monoterpenes due to the presence of the free hydroxyl group, hydrophobicity, and phenol moiety [7]. Essential oils rich in monoterpenes, such as Carvacrol, have demonstrated promising antileishmanial properties due to their lipophilic nature and ability to parasitize membranes and inhibit metabolic pathways [9]. Studies have shown greater efficacy of Car against *Leishmania* spp. compared to other compounds, such as thymol and linalool [6]. Other studies have shown an increase in the potential against *Leishmania* spp. strains of the combination of Car with ascaridol and Car with limonene [10]. However, their clinical translation is limited by poor solubility, ease of oxidation and volatilization and stability, highlighting the need for advanced drug delivery systems [10, 11, 12].

A promising therapeutic strategy to overcome these limitations is the incorporation of Car into nanocarrier systems [10]. Nanotechnology has emerged as an innovative approach to enhance drug bioavailability, reduce toxicity, and improve targeted delivery, as seen in the successful integration of nanomedicine with natural product‐based therapies [13]. Among various nanocarriers, NPChi offer several advantages, including biocompatibility, biodegradability and the ability to facilitate cellular uptake, making them suitable for controlled drug release applications [14]. NPChi are particularly useful due to their small size, providing a large surface‐to‐volume ratio, and physicochemical properties that may differ from those of their bulk counterparts. NPChi are formed from a natural biopolymer, they can be readily functionalized with drugs, RNA, DNA, and other molecules to achieve a desired outcome in vivo [15].

Previous studies have explored the potential of nanoparticles (NPs) for targeted drug delivery, with magnetic microrobots and polymeric nanocarriers showing enhanced therapeutic efficacy against infectious agents [16]. These advantages may make these systems promising for encapsulating Car with antileishmanial effect. NPChi are used for the encapsulation of the main essential oil components, such as Car and cinnamaldehyde, and stand out for their low toxicity, good stability in biological environment, promoting the delivery of active compounds through the membrane of macrophages infected by *Leishmania* spp. [10, 17].

Finally, among the varieties of NPChi, the ionic gelation technique has been the most used, as it is a method considered effective, simple, fast, non‐toxic and free of organic solvents [18].

Thus, NPChi may be considered promising as a delivery system for drugs and anti‐leishmanial bioactive molecules. Therefore, this study aims to develop a chitosan‐based NP system encapsulating Car and evaluate its efficacy against *L. infantum* in vitro, with the aim of improving the biological activity of Car and reducing hemotoxicity.

## 2 Results and Discussion

### 2.1 Preformulation of Chitosan NPs

The preparation of the NPs was performed by the technique of ionic gelation. At the end of preparation, the samples had different macroscopic aspects with the different concentrations of Chi and TPP. The samples were visually characterized as transparent and stable formulations (NPChi2, NPChi3 and NPChi6) and turbid with the formation of precipitates (NPChi1, NPChi4 and NPChi5). Chi:TPP solutions in a mass ratio of 3:1 obtained slightly opaque NPs and Chi:TPP solutions in a mass ratio 1:1 obtained NPs turbid with and agglomerates [18].

### 2.2 Physicochemical Characterization of Chitosan NPs

#### 2.2.1 Size of Particle, Polydispersity Index and Zeta Potential

The results of the average hydrodynamic diameter, polydispersity index (PDI) and zeta potential of the six samples analyzed were determinate (Table 1). It was found that increasing the concentrations of the TPP solutions compared to the Chi solution for the preparation of the NPChi1 and NPChi4 samples resulted in formulations with smaller average particle sizes and high PDI values. It was also possible to analyze that when the formulations had Chi:TPP concentrations in the mass ratio 1:1 (NPChi1 and NPChi5), they had particle sizes greater than 1000 nm and PDI greater than 0.4. The mass ratios of Chi:TPP at 1:1 resulted in turbid colloidal formulations with agglomerates, characterized by high particle sizes and PDI, and reduced zeta potential [18]. Supposedly, it is understood that when the concentrations of Chi:TPP are in the same proportion, they are not able to form stable and homogeneous NPs with smaller sizes.

**TABLE 1:** Particle size, PDI and zeta potential of the NPChi 1‐6 formulations.

Nanoformulation	Concentration of solutions (%*w/v*)	Size (nm)	PDI	Zeta potential (mV) ζ
NPChi1	0.05% (Chi) 0.05% (TPP)	1794.8±178.83	0.476	+6.04±0.46
NPChi2	0.1% (Chi) 0.05% (TPP)	89.43±0.77	0.168	+12.8±2.17
NPChi3	0.2% (Chi) 0.05% (TPP)	161.26+0.37	0.475	+36.7±1.35
NPChi4	0.05% (Chi) 0.1% (TPP)	362.63±170.99	0.800	+4.9±0.29
NPChi5	0.1% (Chi) 0.1% (TPP)	1536.6±27.53	0.609	+4.1±0.22
NPChi6	0.2% (Chi) 0.1% (TPP)	147.36±0.66	0.228	+13.1±0.85

Abbreviations: Chi, chitosan; NPChi1, chitosan nanoparticles prepared by at concentration of 0.05% *w/v* and TPP in at concentration of 0.05% *w/v*; NPChi2, chitosan nanoparticles prepared by at concentration of 0.1% *w/v* and TPP in at concentration of 0.05% *w/v*; NPChi3: chitosan nanoparticles prepared by at concentration of 0.2% *w/v* and TPP in at concentration of 0.05% *w/v*; NPChi4: chitosan nanoparticles prepared by at concentration of 0.05% *w/v* and TPP in at concentration of 0.1% *w/v*; NPChi5: chitosan nanoparticles prepared by at concentration of 0.1% *w/v* and TPP in at concentration of 0.1% *w/v*; NPChi6: chitosan nanoparticles prepared by at concentration of 0.2% *w/v* and TPP in at concentration of 0.1% *w/v*; TPP, tripolyphosphate.

**FIGURE 1:**
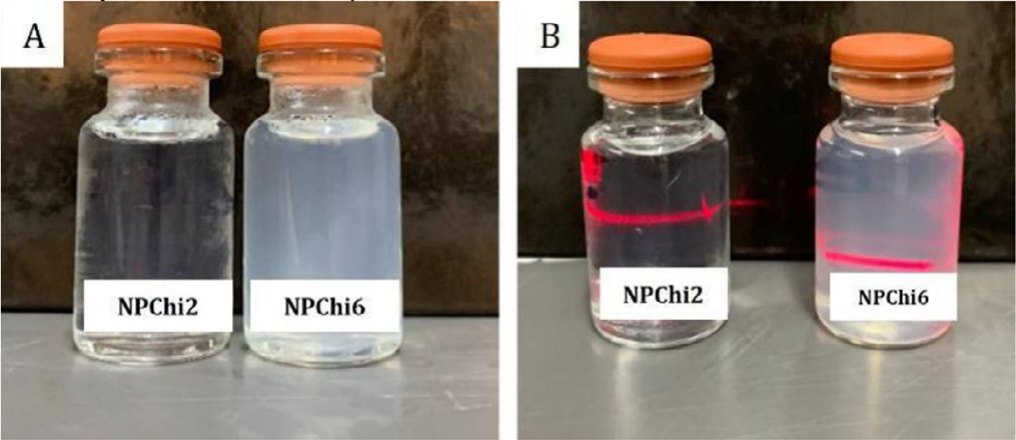
(A) Macroscopic appearance of NPChi2 and NPChi6. (B) Visualization of the Tyndall effect in samples of NPChi2 and NPChi6.

In comparison with the previous ones, the NPChi2 and NPChi6 (Figure 1A) samples with Chi:TPP concentrations in the mass proportions of 2:1 had particle sizes between 80 and 150 nm, and PDI smaller than 0.3. Colloidal systems with particle size smaller than 200 nm are ideal for treating diseases, as they allow the circulation of nanostructures through blood vessels, including capillaries [18, 19]. Nanoformulations with PDI <0.3 nm are characterized as monodisperse systems, that is, systems characterized by a greater uniformity of distribution of the NPs [18, 20]. These results of average diameter and PDI of the NPs indicate promising systems for drug encapsulation.

The Tyndall effect is characteristic of the formation of colloidal suspensions, which corresponds to the scattering of light provided by a dispersed system when irradiated by a light beam [21]. The dispersion of nanometric‐scale particles in a colloidal solution scatters laser light, forming a coherent light beam, which provides us with a rapid technique for characterizing nanometric colloidal systems [21]. Evidence of the presence of NPs in the NPChi2 and NPChi6 formulations was observed by visualizing the Tyndall effect in the samples, which can be seen in the path of the light due to the dispersion of particles present in the formulations (Figure 1B).

By analyzing the zeta potential, the surface charge of NPChi showed a positive, being determined by the degree of neutralization of the ─NH3^+^ groups by the polyanionic groups of sodium tripolyphosphate [20, 22]. This phenomenon justifies the increase of the zeta potential due increasing concentration of Chi, because the availability of ─NH3+ groups depend on the interactions between the protonated ─NH3^+^ groups of Chi and the phosphates of TPP [23]. Thus, the higher the concentration of Chi, the more ─NH3^+^ groups will be available in the medium and the greater the zeta potential of the sample.

The NPs were moderately stable formulations were obtained, considering that nanostructured systems with surface charges of ±30 mV are less susceptible to agglomeration and destabilization forces [19]. Thus, evaluating the macroscopic aspects and physicochemical parameters such as, less mean particle size, PDI <0.3 and zeta potential positive of the formulations obtained, the formulations NPQui2 and NPQui6 were selected to encapsulate Car.

#### 2.2.2 Formulation of Chitosan Nanoparticles Containing Carvacrol

The formulations of NPChi containing carvacrol are demonstrated in (Table 2) shows the results of the physicochemical characterizations. Particle size, PDI, zeta potential are fundamental parameters to determine the stability and potential of formulations for application in vivo and clinical tests [20]. In the sense, it was observed that the increase in the concentration of Car resulted in NPs with larger sizes and PDI, and positive surface charges (Table 2).

**TABLE 2:** Particle size, PDI and zeta potential of NPCar1–4.

Sample	Concentration of solutions (%*w/v*)	Ration concentration of Chi:Car *w/w*	Size (nm)	PDI	Zeta potential (mV) ζ
NPCar1	0.1% (Chi) 0.05% (TPP)	1:1	144.9±1.7	0.224	+15.7±1.01
NPCar2	0.1% (Chi) 0.05% (TPP)	1:2	245.9±4.24	0.175	+15.4±0.75
NPCar3	0.2% (Chi) 0.1% (TPP)	1:1	679±75.61	0.390	+14.9±0.60
NPCar4	0.2% (Chi) 0.1% (TPP)	1:2	699.5±99.41	0.428	+13.2±0.90

Abbreviations: NPCar 1, chitosan polymeric nanoparticles containing 1 mg/mL carvacrol; NPCar 2, chitosan polymeric nanoparticles containing 2 mg/mL carvacrol; NPCar 3, chitosan polymeric nanoparticles containing 2 mg/mL carvacrol; NPCar 4, chitosan polymeric nanoparticles containing 4 mg/mL carvacrol.

**FIGURE 2:**
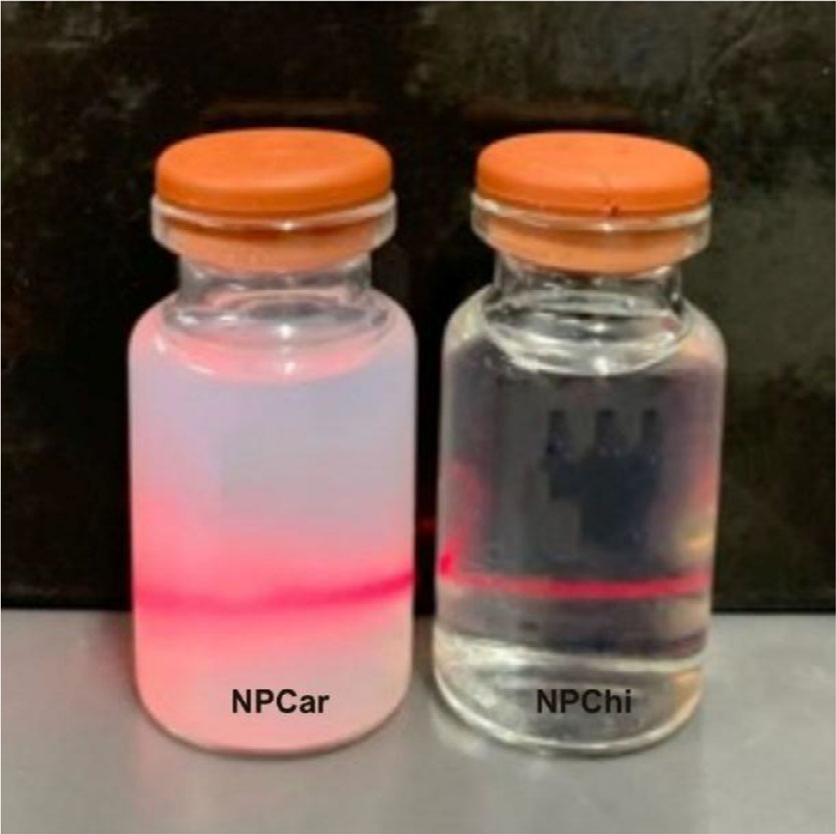
Samples of the nanoformulation of chitosan (NPChi) and chitosan nanoparticles containing Car (NPCar).

Observing the physical‐chemical parameters, the NPCar1 and NPCar2 samples exhibited particle sizes less than 250 nm, PDI less than 0.3 and zeta potential greater than +15, while it is noted that NPCar3 and NPCar4 showed particle sizes in the range of 600 nm, PDI around 0.4 and zeta potential less than +15. In this case, the increase in the amount of Car used was reflected in the increase in the size of the NPs. The PDI values ​​showed the homogeneity of the formulation and indicate a monodisperse suspension, regardless of the ratios used, with PDI values ​​between 0.3 and 0.5, similar to studies by Mondéjar‐López et al. [24]. These results demonstrate that NPCar1 formulation is more homogeneous, stable, and more suitable for delivery to the bloodstream and for the treatment of leishmaniasis, since studies using NPs <200 nm for drug delivery had better results in terms of efficacy for the treatment of leishmaniasis. Smaller NPs can penetrate tissues and cells more easily, increasing the drug concentration at the infection site. Enhances Carvacrol's bioavailability [12, 25]. Thus, considering the activity against promastigote forms of *L. infantum*, after physicochemical characterization analyses, the NPCar1 nanosystem was selected for the following in vitro biological test (Figure 2).

The encapsulation efficiency (EE%) of the NPCar1 formulation was around 51.93%. These results are similar to those in the literature, where chitosan NPs with CAR in a 1:1 ratio had an EE% of 33.4% [24]. The low EE% is due to the fact that some of the molecules escape from the chitosan polymer matrix during the formulation process [24].

#### 2.2.3 Evaluation of the Anti‐Leishmania Activity In Vitro

In the anti‐leishmanial activity, free Car exhibited potential to inhibit the growth of promastigote forms at the concentrations tested (Table 3). Car activity had already been demonstrated in the literature against promastigote strains of *L. infantum* and *L. chagasi* [6, 26]. Thus, these studies consolidate the anti‐leishmanial potential of Car.

**TABLE 3:** In vitro evaluation of anti‐Leishmania activity, effect of cytotoxicity on human erythrocytes and selectivity index (SI_rb_ = HC_50_ /IC_50_).

Sample	IC_50_ (µg/mL)	HC_50_ (µg/mL)	SI_rb_
NPChi	4.51±0.38	> 50	> 11.08
NPCar	2.659±0.26^*^	> 50	> 18.86
Car	11.86±0.31	0.098	0.081
AmB	1.42±0.49	1.94±0.16	1.36

Abbreviations: AmB, Amphotericin B; HC_50_, the concentration needed to cause 50% hemolysis of human red blood cells; IC_50_, 50% of the growth of promastigote forms; NPCar, chitosan polymeric NPs containing carvacrol; NPChi, chitosan polymeric NPs; SI_rb_, selectivity index for erythrocytes.

* Significant difference *p < 0.05* between NPCar and Car.

NPChi showed inhibition of *L. infantum* than free Car (Table 3) proving the biological effect of natural polymers such as Chi in the form of NPs [27]. This is an unprecedented study of the encapsulation of carvacrol in chitosan NPs for the treatment of leishmaniasis. The NPChi presented an IC_50_ of 4.519 ± 0.38 µg/mL, whereas the NPCar presented an IC_50_ of 2.659 ± 0.26 µg/mL. NPCar compared to the free Car reduced the IC_50_ value by 4 times, demonstrating prove the synergism between the NPChi and Car to inhibit the growth of *L. infantum*, in addition to demonstrating the potentialization of leishmanicidal activity using polymeric nanostructures [20, 23, 24].

By comparing NPCar IC_50_ of 2.659 µg/mL with AmB of 1.42 µg/mL, it is possible to highlight that although amphotericin B may be more potent in terms of inhibitory concentration, nanotechnology products can offer other important advantages such as lower toxicity, greater efficacy and a better pharmacokinetic profile.

The literature lacks studies that investigate in more detail the mechanism of action of free carvacrol and NPs on *L. infantum*. It is suggested that the lipophilic nature of Car allows it to act on the cell membrane, making it impermeable to protons and ions, affecting the parasite's enzymatic functions, as well as mitochondria and other cell structures [28]. In addition, encapsulation in NPs allows Car to be delivered with specific targeting [23]. It was shown that oregano essential oil containing carvacrol, thymol, y‐terpinene, p‐cymene and β‐caryophyllene as its main components acted on the promastigote forms of *L. amazonensis*, triggering a combination of autophagic, apoptotic and necrotic events [29]. Studies have shown that oregano essential oil and silver NPs induced morphological, ultrastructural and biochemical changes in *L. amazonensis* promastigotes, revealed by electron microscopy, suggesting a mechanism of cell death similar to apoptosis. Analyses using phosphatidylserine, annexin V and propidium iodide confirmed that the mechanism of action of treatment with this combination is late apoptosis [30]. These results suggest that the NPCar formulation is a promising candidate for future studies with focus in the treatment of VL.

#### 2.2.4 Analysis of Hemolysis in Human Erythrocytes

The hemolytic assay made it possible to determine the HC_50_ for NPChi, NPCar, Car and AmB evidencing the high toxicity of Car and AmB and the absence of toxicity of NPChi and NPCar at the concentrations tested (Table 3). These results indicate that NPChi and NPCar are considered non‐hemolytic, and NPCar was significantly less hemolytic than free Car. The encapsulation reduces concentration of free carvacrol thus the chitosan matrix acts as a physical barrier and slows the release, protecting the carvacrol from direct contact with the membrane [14]. From the above results, it can be concluded that NPCar is a safe and hemocompatible system, and this may be related to the biological nature of the Chi polymer [31]. Thus, the reduction in toxicity observed in this study after Car encapsulation consolidates nanotechnology as a promising therapeutic approach in the treatment of parasitic infections [32].

In addition to cytotoxicity in erythrocytes, the erythrocyte selectivity index (SI_rb_) of NPChi, NPCar, Car, AmB was calculated, and it is noted that the SI_rb_ for NPChi and NPCar were higher compared to Car and AmB. (Table 3). The SI_rb_ allows the differentiation between a general and selective activity of a compound for parasites and provides an indication of safety for the administration of drugs in the control of infections in humans. Furthermore, compounds that present a high SI are indicated for in vivo studies in experimental models [23]. Thus, Car encapsulated in chitosan NPs presents a high degree of selectivity for the parasite, being a promising compound for the treatment of VL.

## 3 Conclusion

In this study, nanostructured systems for encapsulating Car were obtained with biodegradable NPs exhibiting characteristics such as NPCar standing out due to its ideal nanometric size and zeta potential, low polydispersity index, and homogeneous particle distribution. In vitro assays suggest the efficacy of NPCar as a nanotechnology therapeutic approach for the elimination of *L. infantum* and that NPCar enhances Carvacrol's bioavailability. Furthermore, it showed leishmanicidal potential, no hemotoxicity, and an adequate selectivity index for the parasite. Finally, the leishmanicidal potential identified in this research encourages further studies with the amastigote forms of *L. infantum*, as well as for understanding the mechanism of action of nanoencapsulated Carvacrol in these cells.

## 4 Experimental Section

### 4.1 Materials

Low molecular weight Chi (50–190 kDa) with a 75%–85% degree of deacetylation and Car (99% natural) were obtained by Sigma‒Aldrich. Glacial acetic acid PA was obtained by Química Moderna. Sodium tripolyphosphate (TPP) PA and Tween 80 were obtained by Dinâmica. Amphotericin B (Cristália, São Paulo, Brazil).

### 4.2 Preformulation Study of Chitosan NPs

The chitosan solutions were prepared at concentrations in 0.05% *w/v*, 0.1% *w/v* and 0.2% *w/v* in acetic acid solution 1% (*v/v*). This mixture was kept under magnetic agitation for 24 h at a temperature of 25^°^C, and soon after this process, the solution was filtered to remove insoluble material.

The biopolymeric NPChi were prepared by the ionic gelation method [23, 33]. To analyze the influence of the Chi:TPP concentration, 6 formulations were prepared using a mixture of solutions at the concentrations of Chi and TPP. Chitosan solutions prepared at concentrations of 0.05% w/v, 0.1% w/v and 0.2% w/v in acetic acid solution 1% (v/v) were used of TPP in distilled water at concentrations of 0.05% w/v and 0.1% w/v.

To prepare 20 mL of each sample, 0.0032 g of Tween 80 was added to 10 mL of chitosan solution at pH 3,5. This mixture was homogenized by magnetic stirring at 600 rpm for 30 min at a temperature of 50°C. Then, under magnetic stirring, 10 mL of the TPP solution was added dropwise to the chitosan solution under stirring at 600 rpm for 40 min at a temperature of 25^°^C. Finally, the dispersion was sonicated in an ultrasonic sonicator (Qsonica Q55, USA) by 3 min at an amplitude of 30 and power of 55 W. The samples were macroscopically analyzed for color, odor, and presence of precipitation and physicochemically analyzed. The formulations with the best results in terms of particle size, PDI and zeta potential were selected to encapsulate carvacrol.

### 4.3 Encapsulation of Carvacrol in Chitosan NPs

The chitosan NPs containing the Car (NPCar) followed the same method of preparation as the NPChi. 10 mL of aqueous solutions of Chi (0.1%–0.2% *w/v*) and Tween 80 (0.0032 g) were kept under magnetic stirring at 600 rpm for 30 min at a temperature of 50°C. Soon after, Car in proportions of 1:1 *w/w* (chi/car) (NPCar1 and NPCar3) and 1:2 *w/w* (chi/car) (NPCar2 and NPCar4) was added to the aqueous solution of chitosan and homogenized using magnetic stirring at 600 rpm for 10 min. Subsequently, 10 mL of TPP solution (0.05%–0.1% *w/v*) was added dropwise under magnetic stirring at 600 rpm for 40 min at a temperature of 25^°^C. Finally, the dispersion was sonicated in an ultrasonic sonicator (Qsonica Q55, USA.) 3 min at an amplitude of 30 and power of 55w.

### 4.4 Encapsulation Efficiency (EE%)

NPCar1 were analyzed for determining the Carvacrol content at 275 nm using a Carvacrol standard curve with concentrations ranging from 6 to 30 µg/mL. Absorbances were obtained by UV/vis spectrophotometry (UV–Vis‐Jasco spectrophotometer (J‐815). The determination of the Entrapment Drug Efficiency (EE%) was performed using the ultrafiltration‐centrifugation technique. Samples (400 µL) were centrifuged at 9000 rpm for 1 h at 25°C. The supernatant was collected and added to 1 mL of methanol, being measured by a spectrophotometer at 275 nm. The Carvacrol EE% was calculated by Equation (1), based on the straight line equation obtained by the standard curve:

(1) $$\%\mathrm{EE}=\frac{\text{Carvacrol Content}\;-\;\text{Unloaded Carvacrol}}{\text{Carvacrol Content}}$$

### 4.5 Size, PDI and Zeta Potential of NPs

Samples of the NPChi formulations (1–6) and the NPCar were subjected to analysis using photon correlation spectroscopy, also known as dynamic light scattering. Values of the mean diameter and zeta potential for NPChi formulations (1–6) and NPCar were obtained using the Zetasizer Nano ZS equipment (Malvern Panalytical, United Kingdom), using water as a dispersing medium with a scattering angle fixed at 90°C and at a temperature of 90°C and under temperature 25°C in triplicate. The results were analyzed using Zetasizer 7.13 software (Malvern Panalytical, United Kingdom).

### 4.6 Parasites and In Vitro Culture

Biological assays for the evaluation of anti‐leishmanial activity of the in vitro NPChi and NPCar tests were performed following the protocol [34]. The parasites used during the study were forms promastigotes of the species *L. infantum* of the lineage IOC579. The promastigote forms of this *Leishmania* species were maintained in vitro in Schneider medium at pH 7 (Sigma‒Aldrich, St, Louis, USA) supplemented with 20% fetal bovine serum (FBS—Cultilab, São Paulo, BRA), 1% antibiotics (200 U/mL penicillin and 0.1 mg/mL streptomycin—Gibco, Br) and 1% human male urine. The *L. infantum* cultures were incubated at 26°C in a biological oxygen demand oven (B.O.D) with weekly passage of cells.

### 4.7 Evaluation of Anti‐Leishmanial Activity on Promastigote Forms of *L. infantum*

The antileishmanial activity of NPChi and NPCar was evaluated using the 3‐(4,5‐dimethylthiazol‐2‐yl)‐2,5‐diphenyltetrazolium bromide (MTT) test [34]. In a 96‐well plate, 100 µL of supplemented Schneider medium, *L. infantum* promastigotes adjusted to 4 × 10^7^ parasites/well. The samples were added to the wells at serial concentrations of 50 to 0.39 µg/mL and, subsequently, the plate was incubated for 72 h in a B.O.D oven at 26°C. After the incubation time, 10 µL of MTT was added and incubated for another 4 h in a B.O.D oven at 26°C, followed by 100 µL of sodium Dodecyl Sulfate 10% (SDS) for dissolution of the formazan crystals, and lastly, it was read to at 540 nm in a spectrophotometer (Biotek model Elx800; Curitiba, PR, Brazil). The results were expressed as values of inhibitory concentration of 50% of the growth of promastigote forms (IC_50_). The negative control was the supplemented Schneider medium, the positive control was performed with the AmB, and the experiment was performed in triplicate.

### 4.8 Analysis of Hemolysis in Human Erythrocytes

The hemolytic activity of NPChi, NPCar, Car and AmB was determined using human erythrocytes (*n* = 3) according to the method described by [35, 36]. This study was approved by the Ethics Committee for Research Involving Human Subjects of the Lauro Wanderley University Hospital/UFPB, Brazil (CAAE: 17813013.8.0000.5183). All contributors to this research participated voluntarily and signed the Informed Consent Form (ICF). Blood was collected (EDTA tubes). After collection, the erythrocytes were diluted in phosphate saline buffer (PBS) adjusting the blood concentration to 5% of red cells. Next, the NPs and AmB were added in serial concentrations from 50 to 0.39 µg/mL, and the isolated Car in serial concentrations of 1 to 0.007 µg/mL. The samples were incubated for 1 h in a CO_2_ demand oven at 37°C, and after the exposure time, the suspensions were centrifuged at 2000 RPM for 10 min at 25°C, and then each supernatant was transferred to other 96‐well flat‐bottomed plates. The hemolytic activity was monitored by measuring the absorbance at 540 nm in a spectrophotometer (Biotek model Elx800; Curitiba, PR, Brazil). The absence (negative control) and 100% hemolysis (positive control) were determined PBS and Triton X‐100, respectively. The experiment was performed in duplicate in three independent experiments. After statistical analysis, the 50% hemolytic concentration (HC_50_) was calculated using *GraphPad Prism* 5.0 software (San Diego, CA).

### 4.9 Statistical Analysis

The IC_50_ and HC_50_ were calculated using *GraphPad Prism* 5.0 software. Statistical analysis using GraphPad Prism 5.0 was performed using nonlinear regression (curve fit), and analysis of variance (ANOVA) was performed followed by Tukey's posttest, taking a value of *p* < 0.05 as the minimum level necessary for statistical significance. The data represent the mean ± standard error (SEM). The experiments were performed in triplicate.

## Conflicts of Interest

The authors declare no conflicts of interest.

## Data Availability

The authors have nothing to report.

## References

[1] WHO . Leishmaniasis (World Health Organization, 2023), https://www.who.int/en/news‐room/fact‐sheets/detail/leishmaniasis/.

[2] a) I. Abadías‐Granado , A. Diago , P. A. Cerro , A. M. Palma‐Ruiz , and Y. Gilaberte , “Leishmaniasis Cutánea y Mucocutánea,” Actas Dermo‐Sifiliográficas (English Edition) 112 (2021): 601–618. 10.1016/j.ad.2021.02.008 33652011

[3] A. Özbilgin , V. Tunali , I. Çavus , et al., “Visceral Leishmaniasis Caused by Leishmania Tropica,” Acta Parasit 68 (2023): 699–704. 10.1007/s11686-023-00695-w 37351773

[4] OPAS . “Leishmanioses: Informe Epidemiológico Das Américas,” Organização Pan‐Americana Da Saúde (2023), https://iris.paho.org/handle/10665.2/59170.

[5] a) A. De Souza , D. S. S. Marins , S. L. Mathias , et al., “Promising Nanotherapy in Treating Leishmaniasis,” International Journal of Pharmaceutics 547 (2018): 421–431. 29886097 10.1016/j.ijpharm.2018.06.018

[6] a) M. R. Youssefi , E. Moghaddas , M. A. Tabari , et al., “ *In Vitro* and *In Vivo* Effectiveness of Carvacrol, Thymol and Linalool Against Leishmania Infantum,” Molecules (Basel, Switzerland) 24 (2019): 2072. 31151304 10.3390/molecules24112072 PMC6600403

[7] a) M. Sharifi‐Rad , E. M. Varoni , M. Iriti , et al., “Carvacrol and human Health: A Comprehensive Review,” Phyto Res 32 (2018): 1675–1687. 10.1002/ptr.6103 29744941

[8] M. Imran , M. Aslam , S. A. Alsagaby , et al. Food Science & Nutrition 10 (2022): 3544–3561. 36348778 10.1002/fsn3.2994 PMC9632228

[9] L. Monzote , M. García , J. Pastor , et al., “Essential Oil From Chenopodium Ambrosioides and Main Components: Activity Against Leishmania, Their Mitochondria and Other Microorganisms,” Experimental Parasitology 136 (2024): 20–26. 10.1016/j.exppara.2013.10.007 24184772

[10] a) J. Pastor , M. García , S. Steinbauer , et al., “Combinations of Ascaridole, Carvacrol, and Caryophyllene Oxide Against Leishmania,” Acta Tropica 145 (2015): 31–38. 25697866 10.1016/j.actatropica.2015.02.002

[11] M. Imran , M. Aslam , S. A. Alsagaby , et al., “Therapeutic Application of Carvacrol: A Comprehensive Review,” Food Sci Nutr 10 (2022): 3544–3561. 36348778 10.1002/fsn3.2994 PMC9632228

[12] a) R. L. D.e Souza , A. G. B. Dantas , C. D.e O. Melo , I. M. Felício , and E. E. Oliveira , “Nanotechnology as a Tool to Improve the Biological Activity of Carvacrol: A Review,” Drug Deliv Sci Technol 76 (2022): 103834.

[13] a) M. Ye , Y. Zhou , H. Zhao , and X. Wang , “Magnetic Microrobots With Folate Targeting for Drug Delivery,” Cyborg and Bionic Systems 4 (2023): 0019. 37223549 10.34133/cbsystems.0019 PMC10202387

[14] a) R. Jha and R. A. Mayanovic , “A Review of the Preparation, Characterization, and Applications of Chitosan Nanoparticles in Nanomedicine,” Nanomaterials (Basel, Switzerland) 13, no. 8 (2023): 1302. 37110887 10.3390/nano13081302 PMC10140956

[15] a) R. M. Rasul , M. Tamilarasi Muniandy , Z. Zakaria , et al., “A Review on chitosan and Its Development as Pulmonary Particulate Anti‐Infective and Anti‐Cancer Drug Carriers,” Carbohydrate Polymers 250 (2020): 116800. 33049807 10.1016/j.carbpol.2020.116800 PMC7434482

[16] S. Chen , Z. Yang , W. Sun , K. Tian , P. Sun , and J. Wu , “TMV‐CP Based Rational Design and Discovery of α‐Amide Phosphate Derivatives as Anti Plant Viral Agents,” Bioorganic Chemistry 147 (2024): 107415. 38701597 10.1016/j.bioorg.2024.107415

[17] a) R. Essid , A. Ayed , K. Djebali , et al., “Anti‐Candida and Anti‐Leishmanial Activities of Encapsulated Cinnamomum Verum Essential Oil in Chitosan Nanoparticles,” Molecules (Basel, Switzerland) 28 (2023): 5681. 37570651 10.3390/molecules28155681 PMC10419485

[18] D. J. Sullivan , M. Cruz‐Romero , T. Collins , E. Cummins , J. P. Kerry , and M. A. Morris , “Synthesis of Monodisperse Chitosan Nanoparticles,” Food Hydrocolloids 83 (2018): 355–364.

[19] a) M. Danaei , M. Dehghankhold , S. Ataei , et al., “Impact of Particle Size and Polydispersity Index on the Clinical Applications of Lipidic Nanocarrier Systems,” Pharmaceutics 10 (2018): 57. 29783687 10.3390/pharmaceutics10020057 PMC6027495

[20] a) L. A. D.e A. Campos , A. F. S. Neto , A. M. L. Scavuzzi , A. C. D.e S. Lopes , N. S. Santos‐Magalhães , and I. M. F. Cavalcanti , “Ceftazidime/Tobramycin Co‐Loaded Chitosan‐Coated Zein Nanoparticles Against Antibiotic‐Resistant and Biofilm‐Producing Pseudomonas aeruginosa and Klebsiella pneumoniae,” Pharmaceuticals 17 (2024): 320. 38543106 10.3390/ph17030320 PMC10974368

[21] J. Zhu , Y. Wei , J. Zhang , S. Qian , Y. Gao , and W. Heng , “Are All Poorly Soluble Drugs Dissolved in Deep Eutectic Solvents True Solutions?,” Journal of Colloid and Interface Science 645 (2023): 813–822. 37172491 10.1016/j.jcis.2023.04.164

[22] Y. Gokce , B. Cengiz , N. Yildiz , A. Calimli , and Z. Aktas , “Ultrasonication of Chitosan Nanoparticle Suspension: Influence on Particle Size,” Colloids Surf A: Physicochem Eng Asp 462 (2014): 75–81.

[23] a) M. Hadidi , S. Pouramin , F. Adinepour , S. Haghani , and S. M. Jafari , “Chitosan Nanoparticles Loaded With Clove Essential Oil: Characterization, Antioxidant and Antibacterial Activities,” Carbohydrate Polymers 236 (2020): 116075. 32172888 10.1016/j.carbpol.2020.116075

[24] M. Mondéjar‐López , A. J. López‐Jimenez , J. C. García Martínez , O. Ahrazem , L. Gómez‐Gómez , and E. Niza , “Comparative Evaluation of Carvacrol and Eugenol Chitosan Nanoparticles as Eco‐Friendly Preservative Agents in Cosmetics,” International Journal of Biological Macromolecules 206 (2022): 288–297. 35240208 10.1016/j.ijbiomac.2022.02.164

[25] P. Prasanna , P. Kumar , S. Kumar , et al., “Current Status of Nanoscale Drug Delivery and the Future of Nano‐Vaccine Development for Leishmaniasis—A Review,” Biomedicine & Pharmacotherapy 141 (2021): 111920. 34328115 10.1016/j.biopha.2021.111920

[26] J. O. De Melo , T. A. Bitencourt , A. L. Fachin , et al., “Antidermatophytic and Antileishmanial Activities of Essential Oils From Lippia gracilis Schauer Genotypes,” Acta Tropica 128 (2013): 110–115. 23850505 10.1016/j.actatropica.2013.06.024

[27] S. Das , S. Ghosh , A. K. De , and T. Bera , “Oral Delivery of Ursolic Acid‐Loaded Nanostructured Lipid Carrier Coated With Chitosan Oligosaccharides: Development, Characterization, *In Vitro* and *In Vivo* Assessment for the Therapy of Leishmaniasis,” International Journal of Biological Macromolecules 102 (2017): 996–1008. 28465178 10.1016/j.ijbiomac.2017.04.098

[28] L. Monzote , G. Geroldinger , M. Tonner , et al., “Interaction of Ascaridole, Carvacrol, and Caryophyllene Oxide From Essential Oil of Chenopodium Ambrosioides L. With Mitochondria in Leishmania and Other Eukaryotes,” Phytotherapy Research 32 (2018): 1729–1740. 29672979 10.1002/ptr.6097 PMC6208284

[29] F. Tomiotto‐Pellissier , B. T. D.a S. Bortoleti , V. M. Concato , et al., “The Cytotoxic and Anti‐Leishmanial Activity of Oregano (Origanum vulgare) Essential Oil: An *In Vitro*, *In Vivo*, and *in Silico* Study,” Industrial Crops and Products 187 (2022): 115367.

[30] A. B. Alves , B. T. Da Silva Bortoleti , F. Tomiotto‐Pellissier , et al., “Synergistic Antileishmanial Effect of Oregano Essential Oil and Silver Nanoparticles: Mechanisms of Action on Leishmania Amazonensis,” Pathogens 12 (2023): 660. 37242330 10.3390/pathogens12050660 PMC10221720

[31] a) S. Jesus , A. P. Marques , A. Duarte , et al., “Chitosan Nanoparticles: Shedding Light on Immunotoxicity and Hemocompatibility,” Frontiers in Bioengineering and Biotechnology 8 (2020): 100. 32154232 10.3389/fbioe.2020.00100 PMC7047933

[32] T. K. Karam , S. Ortega , T. Ueda Nakamura , R. Auzély‐Velty , and C. V. Nakamura , “Development of Chitosan Nanocapsules Containing Essential Oil of Matricaria Chamomilla L. for the Treatment of Cutaneous Leishmaniasis,” International Journal of Biological Macromolecules 162 (2020): 199–208. 32565304 10.1016/j.ijbiomac.2020.06.149

[33] L. Keawchaoon and R. Yoksan , “Preparation, Characterization and *In Vitro* Release Study of Carvacrol‐Loaded Chitosan Nanoparticles,” Colloids and Surfaces. B, Biointerfaces 84 (2011): 163–171. 21296562 10.1016/j.colsurfb.2010.12.031

[34] F. S. Almeida , G. L. S. Sousa , J. C. Rocha , et al., “ *In Vitro* Anti‐Leishmania Activity and Molecular Docking of Spiro‐Acridine Compounds as Potential Multitarget Agents Against Leishmania Infantum,” Bioorganic & medicinal chemistry letters 49 (2021): 128289. 34311084 10.1016/j.bmcl.2021.128289

[35] J. Da Câmara Rocha , K. A. Da Franca Rodrigues , P. L. Do Nascimento Néris , et al., “Biological Activity of Morita‐Baylis‐Hillman Adduct Homodimers in *L. infantum* and *L. amazonensis*: Anti‐Leishmania Activity and Cytotoxicity,” Parasitology Research 118 (2019): 3067–3076. 31392413 10.1007/s00436-019-06403-w

[36] R. Silva‐Carvalho , J. Fidalgo , K. R. Melo , et al., “Development of Dextrin‐Amphotericin B Formulations for the Treatment of Leishmaniasis,” International Journal of Biological Macromolecules 153 (2020): 276–288. 32145228 10.1016/j.ijbiomac.2020.03.019

